# An efficient analytic approach in genome-wide identification of methylation quantitative trait loci response to fenofibrate treatment

**DOI:** 10.1186/s12919-018-0152-7

**Published:** 2018-09-17

**Authors:** Jiayi Wu Cox, Devanshi Patel, Jaeyoon Chung, Congcong Zhu, Samantha Lent, Virginia Fisher, Achilleas Pitsillides, Lindsay Farrer, Xiaoling Zhang

**Affiliations:** 1 Department of Genetics and Genomics, Boston University, 72 E Concord St, Boston, MA 02118 USA; 2 Department of Bioinformatics, Boston University, 72 E Concord St, Boston, MA 02118 USA; 3 Department of Biostatistics, Boston University School of Public Health, Boston University, 715 Albany St, Boston, MA 02118 USA; 4 Section of Biomedical Genetics, Boston University, 72 E Concord St, Boston, MA 02118 USA

## Abstract

**Background:**

The study of DNA methylation quantitative trait loci (meQTLs) helps dissect regulatory mechanisms underlying genetic associations of human diseases. In this study, we conducted the first genome-wide examination of genetic drivers of methylation variation in response to a triglyceride-lowering treatment with fenofibrate (response-meQTL) by using an efficient analytic approach.

**Methods:**

Subjects (*n* = 429) from the GAW20 real data set with genotype and both pre- (visit 2) and post- (visit 4) fenofibrate treatment methylation measurements were included. Following the quality control steps of removing certain cytosine-phosphate-guanine (CpG) probes, the post−/premethylation changes (post/pre) were log transformed and the association was performed on 208,449 CpG sites. An additive linear mixed-effects model was used to test the association between each CpG probe and single nucleotide polymorphisms (SNPs) around ±1 Mb region, with age, sex, smoke, batch effect, and principal components included as covariates. Bonferroni correction was applied to define the significance threshold (*p* < 5.6 × 10^− 10^, given a total of 89,217,303 tests). Finally, we integrated our response-meQTL (re-meQTL) findings with the published genome-wide association study (GWAS) catalog of human diseases/traits.

**Results:**

We identified 1087 SNPs as *cis* re-meQTLs associated with 610 CpG probes/sites located in 351 unique gene loci. Among these 1087 *cis* re-meQTL SNPs, 229 were unique and 6 were co-localized at 8 unique disease/trait loci reported in the GWAS catalog (enrichment *p* = 1.51 × 10^− 23^). Specifically, a lipid SNP, rs10903129, located in intron regions of gene *TMEM57*, was a re-meQTL (*p* = 3.12 × 10^− 36^) associated with the CpG probe cg09222892, which is in the upstream region of the gene *RHCE,* indicating a new target gene for rs10903129. In addition, we found that SNP rs12710728 has a suggestive association with cg17097782 (*p* = 1.77 × 10^− 4^), and that this SNP is in high linkage disequilibrium (LD) (R^2^ > 0.8) with rs7443270, which was previously reported to be associated with fenofibrate response (*p* = 5.00 × 10^− 6^).

**Conclusions:**

By using a novel analytic approach, we efficiently identified thousands of *cis* re-meQTLs that provide a unique resource for further characterizing functional roles and gene targets of the SNPs that are most responsive to fenofibrate treatment. Our efficient analytic approach can be extended to large response quantitative trait locus studies with large sample sizes and multiple time points data.

## Background

Genome-wide association studies (GWASs) have discovered hundreds of genetic loci associated with blood lipid levels [1]. Fenofibrate is a widely used medicine to treat high levels of cholesterol and triglycerides. Because treatment response is heterogeneous and heritable, several recent studies have identified genetic determinants of the observed heterogeneity in fenofibrate effects on lipid levels [2] and systemic inflammation involved in lipid metabolism [3, 4]. However, the functional mechanism underlying these findings is largely unknown.

Epigenetic changes such as DNA methylation alteration contribute to phenotypic differences within and between individuals. The study of genetic effects on methylation quantitative trait loci (meQTLs) has emerged to help dissect regulatory mechanisms underlying these GWAS associations [5, 6]. However, the genetic components that determine the nature and strength of meQTL treatment response (re-meQTL) has not yet been studied. Using the GAW20 genotype and methylation data obtained before and after fenofibrate treatment of the same subjects, we conducted a re-meQTL analysis to identify common single nucleotide polymorphisms (SNPs) associated with methylation changes. This study is the first genome-wide examination of genetic drivers of methylation variation in treatment response, providing a unique resource for the community.

In addition to the re-meQTL analysis, we developed an efficient analytic approach that significantly reduced the computational burden. The computation of genome-wide meQTL analysis is known to be intensive, especially for re-meQT study which requires more than one time-point data. In this work, we analyzed the pre- and post- treatment methylation data in one model. We believe our approach is more efficient and improves on the recently reported analysis of response-expression QTLs (re-eQTLs) in which different time-point data were first analyzed separately, then the re-eQTLs were defined based on the effect size difference between post stimulus and basal levels [7].

## Methods

### Sample demography

The original samples are from the Genetics of Lipid Lowering Drugs and Diet Network (GOLDN) study (details at https://dsgwebwp.wustl.edu/labs/province-lab/goldn/), which aimed to characterize the genetic impact on response to interventions that affects triglycerides (TGs) [8]. The GOLDN study was done through a collaboration of 6 university-based medical centers and all participants were whites of European ancestry. Our study only used GAW20 real data, which contains participants in response to 4 visits in 3 weeks for an open-label clinical trial of fenofibrate 160 mg. Data from visits 1 and 2 reflected the basal levels for participants before the fenofibrate treatment, whereas visits 3 and 4 were collected after fenofibrate treatment. For our re-meQTL analysis, we included 429 subjects (male = 221, female = 208) from 140 families with genotype and both pre- (visit 2) and posttreatment (visit 4) methylation data available.

### Sample quality control

The methylation of the whole blood was measured by two different probes on the methylation chip, they were adjusted using a beta-mixture quantile normalization (BMIQ) to fit the beta values of Type II design probes of Illumina 450 K into a statistical distribution characteristic of Type I probes [9]. To avoid bias, cytosine-phosphate-guanine (CpG) sites with SNP markers underneath that could be compromised by genotype [10], cross-reactive probes possibly co-hybridizing to alternate genomic sequences [11], and polymorphic probes were removed according to the procedures outlined in Price et al. [10] and Chen et al. [11]. Only subjects with genotype and both post- and pre-treatment methylation data that have complete age, sex, center, and smoking information were included in the analysis (*n* = 429). The SNP genotypes were first filtered to include only *cis* SNPs to each CpG site, and then filtered to only include common variants with a minor allele frequency (MAF) > 0.05. The principal components (PCs) of the genotypes were computed using the GENESIS R package to fit PCs on a maximal unrelated subset and then project all samples to those PCs.

### Association analysis of identifying *cis* re-meQTLs

*Cis* re-meQTL association analysis was done with 208,449 CpG sites, where SNPs within ±1 Mb around the CpG probe were tested in an additive linear mixed-effects model. Log transformed post−/pretreatment methylation values were regressed on SNP genotypes (coded as 0, 1, 2) after adjusting for age, sex, batch effect, smoking status, and first 10 PCs using the *lmekin* function from the coxme R package with pedigree structure as random effects. For a given CpG probe, the model can be explained as below for a SNP within 1 Mb around the CpG:

$$ {\mathrm{Y}}_{\mathrm{i}}={\mathrm{X}}_{\mathrm{i}}\ \upbeta +{\mathrm{U}}_{\mathrm{i}}{\upgamma}_{\mathrm{i}}+{\upvarepsilon}_{\mathrm{i}} $$

in which, *Y*_*i*_ is log_10_ (post/pre) values of cluster/family *i*; *γ*_*i*_ represent the estimates of random effects for pedigree structure that is based on known pedigree relationships; *β* represent the estimates of fixed effects as the following:

$$ {\beta}_0+{\sum}_{j=1}^{10}{\beta j}^{\ast } PCj+{\beta_{11}}^{\ast } Age+{\beta_{12}}^{\ast } Sex+{\beta_{13}}^{\ast } Batch+{\beta_{14}}^{\ast } Smoke+{\beta_{15}}^{\ast } SNP $$

The Bonferroni correction was applied to define statistical significance, resulting in *p* < 5.6 × 10^− 10^, given a total of 89,217,303 tests. A total of 208,449 CpG sites were tested, so 89,217,303 is the summation of all the SNPs that are within ±1 Mb around each CpG site. The average number of SNPs per CpG site is 428 (89,217,303/208,449). Regional plots of all significant SNPs associated with CpGs were generated using LocusZoom.

### Enrichment analysis

Identified *cis* re-meQTL SNPs (*p* < 5.6 × 10^− 10^) from the above association tests were compared with disease−/trait-associated SNPs in the National Human Genome Research Institute–European Bioinformatics Institute (NHGRI-EBI) GWAS Catalog (downloaded on 01/09/2017) [12] by rsID. A binomial test was conducted to test the enrichment of our unique *cis* re-meQTL SNPs in the GWAS catalog. Regional plots were generated for *cis* re-meQTL SNPs that were also found to be associated with lipid traits in the GWAS catalog using our re-meQTL results and published lipid GWAS results [1] for comparison using LocusZoom.

## Results

### CpG-SNP association

**Fig. 1:**
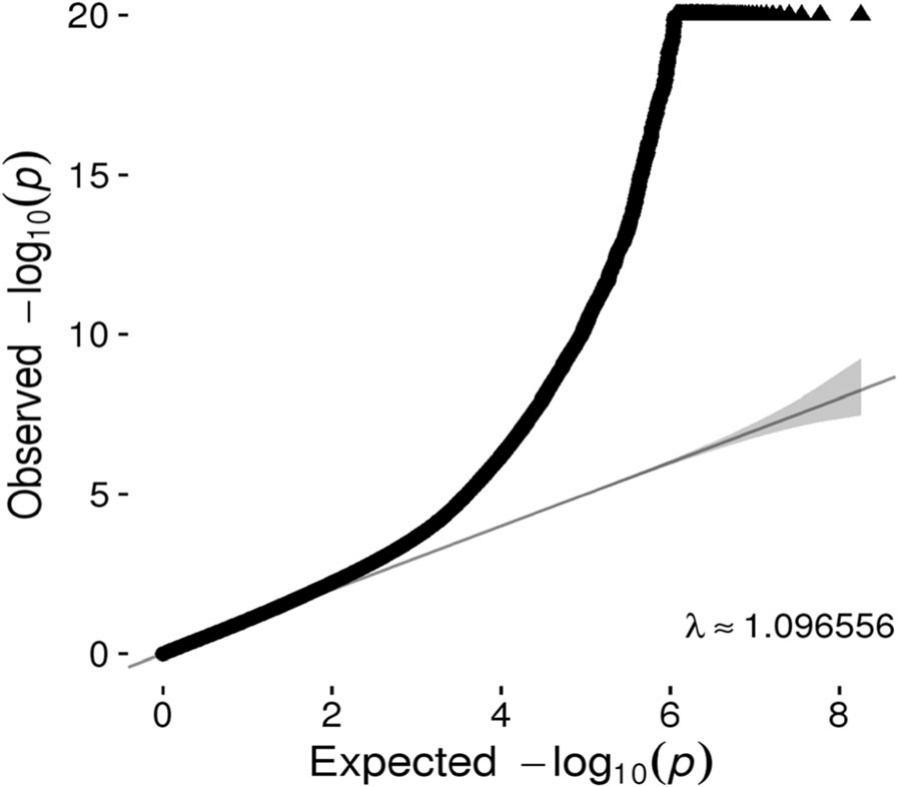
Q-Q plot of *cis* re-mQTLs, Expected *p* value of a null distribution was plotted on a negative log scale (*black line*) and the *p* values of the re-mQTLs was plotted on a negative log scale (*black dots*).The 95% confidence interval of the observed *p* value is shown in gray. The genomic inflation lambda is 1.097

Quantile–quantile (Q-Q) plots (Fig. 1) for SNPs with a MAF > 5% did not show evidence of substantive overdispersion/genomic inflation in our CpG-SNP association test (λ = 1.097), especially considering that SNPs within a ± 1 Mb of each CpG site were analyzed so that some of the correlated SNPs within that region were analyzed more than once. Because sample relatedness was controlled as a random effect in the linear mixed-effects model, a model that has proven to successfully control the genome-wide error rate [12], we optimized for controlling of the relatedness. Population structure also was controlled by adding PCs to the model.

**Fig. 2:**
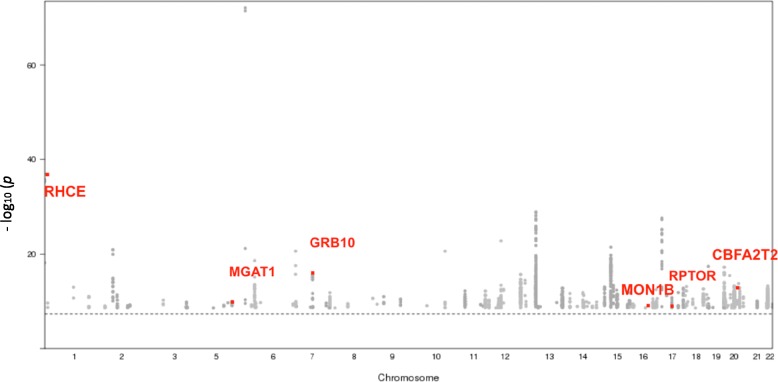
The Manhattan plot of *cis* re-mQTLs (*p* < 5.6 × 10^− 10^) identified in this study with GWAS SNPs reported in the National Human Genome Research Institute (NHGRI) Catalog (highlighted in red)

At a *p* value threshold of 5.6 × 10^− 10^, 1087 SNPs located in 351 unique gene regions were identified as *cis* re-meQTLs associated with 610 CpG sites. Among these 1087 *cis* re-meQTL SNPs, 229 are unique. Figure 2 highlights in red the *cis* re-meQTL SNPs with 6 loci found in the GWAS catalog. In addition, the most significant *cis* re-meQTL, rs3733749, is associated with cg00514575 (*p* = 8.06 × 10^− 73^) and is upstream of *MGAT1*, a gene related to TG/lipid accumulation [13], suggesting that a novel genetic variant might regulate a nearby lipid gene, *MGAT1*, through DNA methylation.

### GWAS catalog look-up and enrichment analysis

**Fig. 3:**
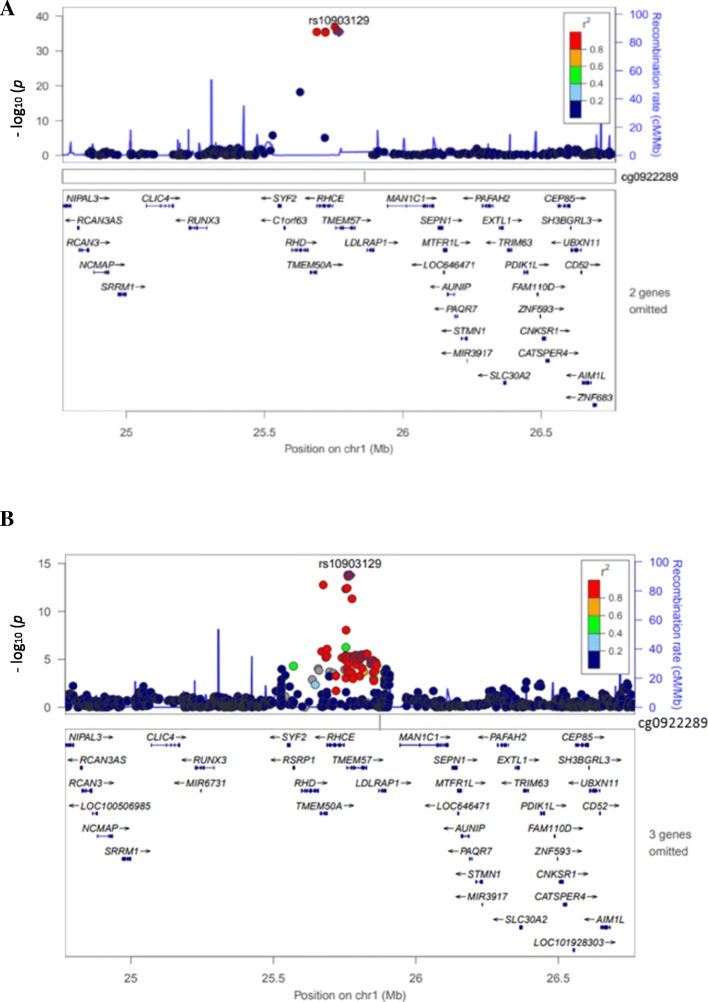
Regional plot of SNP rs10903129. **a** Associated with cg09222892 probe (chr1: 25861512–25,861,513). **b** Associated with total cholesterol GWAS results in which the SNP is mapped to *TMEM57*

Among 1087 *cis* re-meQTLs, 6 unique SNPs were co-localized at 8 unique disease/trait loci reported in the NHGRI GWAS catalog (Table 1).As reported, a lipid SNP, rs10903129, was associated with total cholesterol (*p* = 7.0 × 10^− 11^), and low-density lipoprotein (LDL) cholesterol (*p* = 2.0 × 10^− 19^) [1]. As Table 1 shows, SNP rs10903129, located in an intron of gene *TMEM57*, is a re-meQTL (*p* = 3.20 × 10^− 36^) associated with cg09222892, which is upstream of the gene *RHCE*, indicating a new function target gene for this reported lipid SNP rs10903129. Figure 3a shows the top re-meQTLrs10903129 results in this region, whereas Fig. 3b displays published LDL GWAS results of the same SNP in this region [1, 14]. Among these 1087 *cis* re-meQTL SNPs, 229 are unique and 6 are also found in the GWAS catalog (enrichment *p* = 1.51 × 10^− 23^), indicating that the re-meQTLs identified in our study are highly relevant to human diseases/traits.

**Table 1:** Top *cis* re-mQTLs that were matched to GWAS catalog

SNPs	CpGsite	CpGgene	meQTL*p* value	CpG and SNP difference (bp)	Disease/trait	Gene in GWAS	SNPfunction
rs10903129	cg09222892	*RHCE*	3.20E-36	−92,575	Cholesterol, total cholesterol, erythrocyte sedimentation rate	*TMEM57*	Intron
rs2237457	cg03466198	*GRB10*	2.82E-16	−34,294	Schizophrenia (treatment resistant)	*GRB10*	Intron
rs6120141	cg14296766	*CBFA2T2*	7.41E-14	− 999,855	Smooth-surface caries	*BPIFA2*	Intron
rs12517906	cg00514575	*MGAT1*	4.80E-11	−127,730	Weight	*OR2Y1-MGAT1*	Upstream
rs1046896	cg12045294	*RPTOR*	1.43E-10	− 239,794	Glycated hemoglobin levels	*FN3KRP*	3′ Untranslated region
rs11150078	cg07648639	*MON1B;SYCE1L*	3.21E-10	− 208,317	Bipolar disorder (body mass index interaction)	*WWOX*	Intron

The most significant SNP from each gene is shown

### Co-localization of re-meQTL with GWAS results of fenofibrate effects

**Fig. 4:**
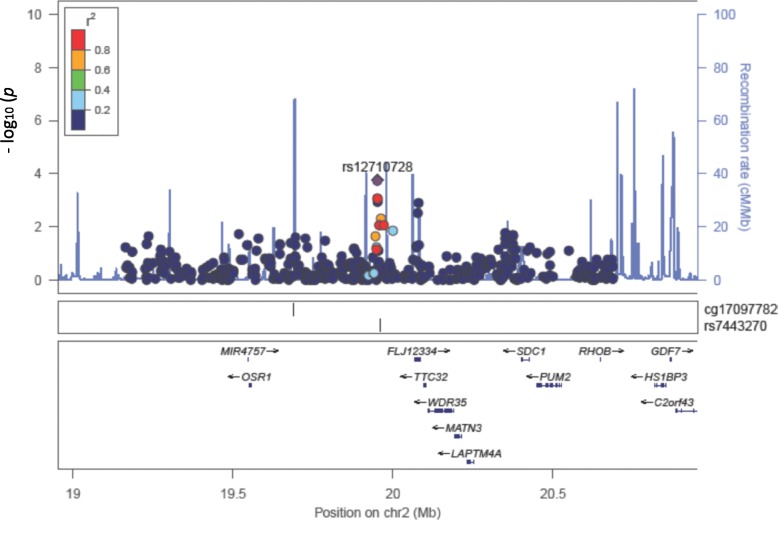
Regional plot of SNP rs12710728–associated cg17097782 probe (annotation: *OSR1*)

Among the GWAS results of fenofibrate effects reported by the GOLDN study, we are interested in whether the *cis* re-meQTLs from our study could serve as epigenetic evidence of how genetic loci associated with fenofibrate treatment can be explained by the variation of methylation changes, and whether there is methylation change variation relevant to the genetic loci associated with fenofibrate effects on phenotype variation such as the changes of lipid or inflammatory biomarkers. As shown in Fig. 4, we found a SNP (rs12710728) that has a suggestive association with a CpG site (cg17097782, *p* = 1.77 × 10^− 4^), which is in high linkage disequilibrium (LD) (R^2^ > 0.8) with the GWAS-reported SNP rs7443270 for TG-lowering effects of fenofibrate (*p* = 5.00 × 10^− 6^) [3].

## Discussion

Epigenetic modifications such as DNA methylation can account for phenotypic differences within and between individuals. Specifically, meQTL studies can help dissect regulatory mechanisms underlying GWAS loci. In this short study, we identified thousands of *cis* re-meQTLs that link to the methylation differences in fenofibrate treatment responses, which could provide genetic and epigenetic determinants for TG level differences between pre- and post-treatment in individuals. As part of our results, we have helped to further validate disease-relevant genes such as *MGAT1* [13] and *RHCE* [15]. In addition, we established a link between meQTL and reported fenofibrate GWAS SNPs via LD. As a result of the coverage of the 450 K Illumina chip, none of the reported fenofibrate GWAS SNPs were found in our genotype data after quality control. However, we did identify a suggestive significant meQTL, rs12710728, which is in high LD with a reported fenofibrate treatment SNP (rs7443270). Our findings provide a unique drug-response–specific meQTL resource for further characterizing the potential functional roles of GWAS SNPs that show response differences to fenofibrate treatment. Finally, as the first response-specific meQTL study of its kind, it will also serve as an important resource for future studies.

Because of the large number of SNPs and CpG sites involved, the computation for meQTL analysis can be quite intensive, especially when looking at both pre- and posttreatment data, or even both *cis* and *trans* SNPs. We believe our novel analytic approach to the computation improves on the method used in the Quach et al. study [7], which focused on response-specific expression quantitative trait locus (eQTL). We used the log difference of the pre- and posttreatment states, which can be more efficient than looking at the basal and posttreatment states separately, as done in the Quach et al. study [7]. In the future, we can further validate our approach by optimizing our pipeline and conducting analysis on pre and post states separately, using an interaction model, as well as analyzing both *cis* and *trans* re-meQTLs.

## Conclusions

In this study, we aim to investigate cis-methylation loci that are responsible for lipid lowering drug fenofibrate treatment. Thousands of *cis* re-meQTLs that link to the methylation differences were identified, which provide genetic and epigenetic determinants for TG level differences between pre- and post- treatment among individuals. As the first response-specific meQTL study of this kind, these results will serve as an important resource for future studies of the potential functional roles of GWAS SNPs that show response differences to fenofibrate treatment.
